# The Effectiveness of Cinacalcet as an Adjunctive Therapy for Hereditary 1,25 Dihydroxyvitamin D_3_-Resistant Rickets

**DOI:** 10.4274/jcrpe.3486

**Published:** 2017-06-01

**Authors:** Ayşehan Akıncı, İsmail Dündar, Meltem Kıvılcım

**Affiliations:** 1 Inönü University Faculty of Medicine, Department of Pediatric Endocrinology, Malatya, Turkey; 2 İnönü University Faculty of Medicine, Department of Developmental and Behavioral, Malatya, Turkey

**Keywords:** Cinacalcet, hereditary vitamin D-resistant rickets, secondary hyperparathyroidism

## Abstract

High doses of oral calcium or long-term calcium infusions are recommended to correct the hypocalcemia and secondary hyperparathyroidism in patients with hereditary 1,25 dihydroxyvitamin D_3_-resistant rickets (HVDRR). Preliminary studies revealed that calcimimetics may be a safe and effective therapeutic choice in children with secondary hyperparathyroidism. Our aim was to observe the efficacy of cinacalcet in the normalization of secondary hyperparathyroidism and hypophosphatemia in two siblings aged 2.5 years and 6 months with HVDRR who did not respond to traditional treatment regimes. Both patients were admitted to the hospital with severe hypocalcemia. They were treated with high doses of calcitriol and calcium infusions intravenously. Secondary hyperparathyroidism was normalized temporarily, but did not improve completely. Cinacalcet (0.25 mg/kg) once a day along with the high doses of oral calcium and calcitriol was added to the treatment schedule. After 3 months, biochemical and radiologic findings reverted to normal. Our findings indicate that cinacalcet is effective in normalizing the hyperparathyroidism and hypophosphatemia in these cases and in improving the bone pathology.

## What is already known on this topic?

Hereditary vitamin D-resistant rickets (HVDRR) is a rare autosomal recessive disease characterized by early-onset severe rickets, alopecia, hypocalcemia, and secondary hyperparathyroidism in the face of an elevated serum 1,25 dihydroxyvitamin D_3_ [1,25(OH)2D_3_] level. Secondary hyperparathyroidism from inadequate calcium absorption in the gut is the underlying pathophysiology for the rachitic changes in HVDRR. The recent availability of the calcimimetic agent cinacalcet enables the suppression of parathyroid hormone secretion through activation of the calcium-sensing receptors.

## What this study adds?

We observed that cinacalcet is effective in the management of secondary hyperparathyroidism and that it improves the biochemical and radiological findings in HVDRR. The calcimimetic agent, cinacalcet, may be considered as an adjunct to high-dose 1,25(OH)2D_3_ (calcitriol) and calcium therapy in the management of children with HVDRR.

## INTRODUCTION

Hereditary vitamin D-resistant rickets (HVDRR) is a rare autosomal recessive disorder caused by mutations in the *vitamin D receptor* (VDR) gene. It is characterized by severe rickets, hypocalcemia, hypophosphatemia, secondary hyperparathyroidism (hPTH), increased serum alkaline phosphatase (ALP) and 1α,25 dihydroxyvitamin D_3_ [1,25(OH)_2_D_3_] levels (1,2). Total or partial alopecia and dermal cysts are encountered in a subset of these pediatric patients. The *VDR* gene is expressed in most tissues of the body, including intestines, kidney, bones, and keratinocyte of hair follicles (3). Alopecia due to the defective *VDR* gene activity within keratinocytes appears in approximately two-thirds of the cases and is considered as a marker of disease severity and response to therapy. There is no standard therapy protocol for patients with HVDRR, and patients with alopecia are usually more resistant to treatment and require high doses of 1,25(OH)_2_D_3_ and also large doses of calcium, either administered orally or intravenously (4,5,6). Most patients with HVDRR require prolonged hospitalization for intravenous calcium therapy in order to maintain normocalcemia. Especially in infants, prolonged hospitalization and high infection risk related to catheter application are a few of the difficulties of this treatment regime. Another risk is unsustained parathyroid hormone (PTH) suppression and related hypophosphatemia and poor healing of the skeletal findings. It is possible to suppress the development of secondary hPTH, normalize serum phosphate levels, and resolve the rachitic changes with cinacalcet, which is a calcimimetic (7). We report the effectiveness of cinacalcet on the normalization of metabolic parameters and radiological healing of rickets in two sisters with HVDRR who had inadequately controlled secondary hPTH despite treatment with oral/or intermittent i.v. calcium infusion and calcitriol in high doses.

## CASE REPORTS

### Case 1

**Figure 1:**
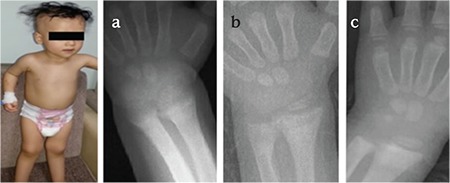
Alopecia and rickets in case 1 1a. Before treatment: Anteroposterior radiography of the patient’s hand demonstrating cupping of the metaphyseal region 1b. After treatment with high dosages of i.v. calcium infusion, wrist x-ray showing partial healing of the rickets findings 1c. The x-ray of the wrist showing progressive healing of rickets while the child was receiving high-dose oral calcium, calcitriol, and cinacalcet

**Figure 2:**
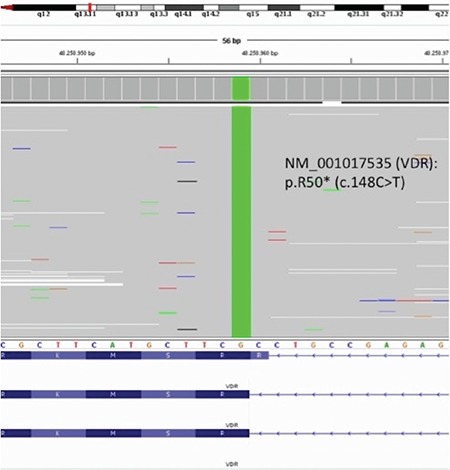
VDR gene analysis performed by using MiSeq next-generation sequencing platform, a Food and Drug Administration-approved diagnostic system (Illumina, San Diego, CA, USA). The gee has 11 exons and NM-001017535(VDR): p.R50* (c.148 C>T) is causing a premature stop codon in exon 5. This variation causes a truncated protein and severe damage on protein function

The proband was a 2.5-year-old female child who was admitted to our hospital for evaluation of her seizures, deformities of lower extremities, failure to thrive, and alopecia. She was born to consanguineous parents as a full-term infant by normal spontaneous vaginal delivery, with a birth weight of 3250 g and a birth length of 50 cm. Her mother had received prenatal care and had taken vitamins regularly. The patient had been almost exclusively breastfed since birth up to 15 months. In addition, she had received 25-hydroxyvitamin D_3_ [25(OH)D_3_] 400 IU per day for one year. At presentation, her height was 83 cm (3-10% p), her weight was 10.7 kg (weight for height: 25% p), and head circumstance was 47 cm (3-97% p). She had rachitic rosary over the chest wall, widening of wrists and ankles, and “X” deformity of lower extremities. Her head appeared disproportionately large, but head circumference was within the normal range with an open anterior fontanel and frontal bossing. She was able to sit without support, but her ability to stand and walk were restricted due to the “X” deformity of the legs. She had frontal bossing, near total alopecia, and sparse eyebrows and eyelashes (Figure 1). Laboratory results revealed normal levels for serum electrolytes, serum albumin, blood urea nitrogen (BUN), and creatinine. Other laboratory results were as follows: serum calcium (Ca): 7.5 mg/dL (8.8-10.8), serum phosphorus (P): 2.3 mg/dL (4.5-5.5), ALP: 2278 U/L (80-220), intact PTH: 1194 pg/mL (10-71 pg/mL), 25(OH)D_3_: 25.2 ng/mL (10-44 ng/mL), 1,25(OH)_2_D_3_: 59 pg/mL (16-65 pg/mL). Serum Ca, P, and ALP were determined by spectrophotometric method (Abbott, Architect C 16.000, IL, USA), intact PTH by chemiluminescent method (Beckman Caulter DxI800), and 25(OH)D_3_ by electro chemiluminescent method (Roche, Cobas e-411). Serum electrolytes, albumin, BUN, and creatinine levels were within normal ranges. Serum 1,25(OH)_2_D_3_ level was in the upper limit of the normal range. The skeletal survey showed generalized osteopenia with advanced features of rickets manifested by cupping and fraying at the metaphyseal ends of long bones of upper and lower extremities (Figure 1a). VDR gene sequence analysis was performed by using MiSeq next-generation sequencing (NGS) platform, a Food and Drug Administration-approved diagnostic system (IIIumina, San Diego, CA, USA), and it was shown as a homozygote stop-codon mutation (c.148C>T) with mutation number: NM-001017535 (Figure 2). This mutation was described previously (8). Parents were heterozygous for the same mutation.

As shown in Table 1, the patient was initially treated with high doses of oral calcium (elemental Ca: 2 g/day), phosphate (1 g/day), and calcitriol [1α-25(OH)D_3_] (2 µg/day). Elemental calcium and calcitriol were subsequently increased to 4 g/day and 6 µg/day, respectively. After 8 weeks of this protocol, serum Ca concentration became close to normal levels, but PTH was not suppressed and ALP was still high, and no radiological improvement was observed. Treatment was continued with high-dose oral calcium and calcitriol. After 6 weeks, the patient was admitted to hospital again with hypocalcemic seizures. During this period, HVDRR was confirmed by VDR gene mutation and intravenous calcium infusion was initiated. Elemental calcium was administrated at 150 mg/kg/day, infused via central line over a period of 10 hours for five days in a month. She has been on this treatment for 4 months. We observed a decrease in PTH and ALP levels and normalization of serum P levels only during the periods of intravenous calcium infusion administration. After 4 steps of i.v. calcium infusion, partial healing in skeletal bone rickets findings were observed (Figure 1b); however, when the calcium infusion was stopped, secondary hPTH, increased ALP levels, and hypophosphatemia developed again and rachitic bone features became more apparent in the radiograms. After 4 months of intermittent calcium infusion, serum PTH and ALP levels decreased to near normal levels and radiological improvements were detected, secondary hPTH was normalized temporarily but not improved completely. After these improvements, intravenous calcium treatment was stopped and oral calcium and calcitriol treatment in high doses was administered again. However, after a while, serum PTH and ALP levels started rising again, serum Ca and P levels tended to decrease. We started cinacalcet (0.25 mg/kg) once a day along with high doses of oral calcium (4 g/day) and calcitriol (6 µg/day) and the cinacalcet dose was incrementally increased based on serum calcium and PTH levels, reaching 0.4 mg/kg/day over the next 2 weeks. We observed a temporary hypocalcemia during cinacalcet therapy. After 4 months of cinacalcet, serum Ca and P levels were within normal limits and we also observed sustained control of serum PTH and ALP levels and healing of the radiological rickets findings (Figure 1c). Treatment has been continued with a decreased dose of calcitriol (2 µg/day) and oral Ca (2 g/day) along with low-dose cinacalcet.

### Case 2

**Figure 3:**
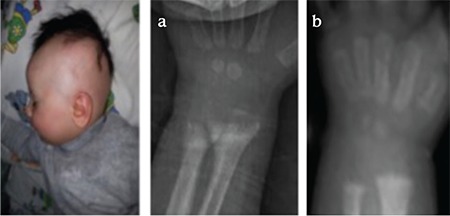
Alopecia and rickets in case 2 3a. Baseline x-rays at the age of 4 months showing changes consistent with rickets in case 2 3b. Bone roentgenogram showing markedly improved signs of rickets with high-dose calcitriol, oral calcium, and cinacalcet

The proband’s sister was a 4-month-old girl with nearly total alopecia since birth (Figure 3). She had no history of hypocalcemic symptoms or seizures. She was taking daily 25(OH)D_3_ (400 IU). At first visit, we have not observed any pathological features at upper and lower extremities. Her length was 63 cm (50% p), weight was 6.5 kg (25% p), and she had no radiological findings of rickets. Laboratory examinations showed normal serum Ca and P levels; however, serum PTH and ALP levels were high. The same mutation of VDR gene which was previously detected in her sister was also found in this patient. In her next visit after 2 months, serum Ca and P levels tended to decrease (Ca: 7.9 mg/dL and P: 2.8 mg/dL), but PTH (480 pg/mL) and ALP (689 U/L) levels were increased (Table 2). Rickets findings were observed radiologically in her elbow joint (Figure 3a). The same treatment protocol which was applied to her sister was also applied to this patient. At first, she was treated with high-dose calcitriol and intermittent calcium infusion; a temporary metabolic and radiological improvement was detected, as in her sister (Table 2). After adding cinacalcet to the treatment schedule, PTH and ALP levels decreased, serum Ca and P levels were sustained within normal levels, and complete radiological healing of rickets was observed (Figure 3b).

Informed consent forms were obtained from the parents of the patients for publication of the cases, including images.

## DISCUSSION

HVDRR results from loss of *VDR* gene function leading to target-organ resistance to 1,25(OH)_2_D_3_ which regulates Ca/P metabolism and bone mineralization. It is associated with severe rickets, secondary hPTH due to hypocalcemia, and hypophosphatemia with or without alopecia. Serum 1,25(OH)_2_D_3_ level is usually elevated. While 25(OH)D_3_ levels were within normal limits in our patients, 1,25(OH)_2_D_3_ levels were in the upper limits of the normal ranges (1,2,3).

Alopecia has been considered as one of the indicators of severe hormone resistance in patients with HVDRR. Though there is genotype-phenotype variability, the severity of alopecia and hormone resistance seems mostly to depend on the different types of VDR mutation in these patients. Several mutations in the *VDR* gene have been identified as the cause of HVDRR. It has been revealed that most of the patients with mutations in the DNA-binding domain have alopecia, whereas patients with mutations in the ligand-binding domain have variable degrees of vitamin D resistance usually not associated with alopecia (1,9,10). In our patients, we identified a stop-codon mutation in the *VDR* gene (c.148C>T) in both sisters as previously described in two sisters with alopecia and HVDRR (8). In these patients, the mutation was transmitted in an autosomal recessive inheritance, and the parents who had the same mutation in heterozygous form were asymptomatic, with no features of metabolic bone disease or alopecia. Our patients had near total alopecia and severe rickets findings on physical examination and X-ray findings similar to those of the two sisters carrying the same VDR mutation, previously reported (8).

In HVDRR, the main metabolic characteristics are severe hypocalcemia-induced secondary hPTH and renal phosphate wasting which prolongs bone healing. Traditional therapy with high-dose oral calcium and calcitriol aims to correct serum Ca concentration to normal levels and to radiological healing of rickets. However, these treatment protocols are usually not able to suppress the secondarily increased hPTH and ALP levels. Sustained hPTH prolongs the bone healing process by stimulating bone turnover and hypophosphatemia. Actually, serum PTH level is suppressed by ionized serum Ca concentrations. But it is difficult to maintain normocalcemia and to suppress PTH level in patients with HVDRR, especially patients with alopecia. For these reasons, some authors proposed HVDRR to be a form of PTH-dependent rickets. High doses of calcium and calcitriol treatment which are required to maintain normocalcemia may lead to vitamin D intoxication, nephrocalcinosis, and kidney damage. Moreover, tertiary hPTH and osteitis cystica fibrosa may develop in some patients with HVDRR (11,12). Some authors recommend parathyroidectomy in patients with HVDRR who have unsuppressed secondary hPTH. It has also been proposed that parathyroidectomy can be considered not only for correction of hypocalcemia but also for PTH suppression to maintain metabolic and radiological improvement in HVDRR. In Case 1, during 2.5 months of high-dose oral calcium and calcitriol therapy, serum Ca concentration reached a near normal level from time to time, but serum P level was lower than normal limits despite phosphorus supplementation, and serum PTH and ALP levels were still extremely elevated. During this period, radiological healing was not observed. Enteral or parenteral calcium infusions are reported to suppress PTH level with metabolic and radiological healing in HVDRR patients with or without alopecia (4,5,6). In our patients, long-term normocalcemia and normalization of hypophosphatemia secondary to unsustained hPTH suppression could not be achieved with high-dose oral calcium and calcitriol treatment. Therefore, we tried treatment with intermittent parenteral calcium infusions. As previously reported, two siblings who had the same mutations in VDR gene were successfully treated with parenteral calcium infusion (8). We observed a decrease in PTH and ALP levels and normalization of serum P levels only during the periods of intravenous calcium infusion. After four-step intermittent calcium infusion (each step covering five days of i.v. calcium infusion in a month), metabolic improvement and partial healing in bone findings was observed; but when the calcium infusions were stopped, secondary hPTH and hypophosphatemia occurred again and rachitic bone features became more apparent in X-rays. Intravenous calcium treatment for a long time is difficult especially in infants because of prolonged hospitalization and increased risk of infection due to central catheter application. For these reasons, alternative therapeutic approaches have been tried to suppress PTH. The recent availability of the calcimimetic agent cinacalcet enables the suppression of PTH secretion through activation of the calcium-sensing receptors. Cinacalcet has been used in adults with primary hPTH or secondary hPTH due to chronic renal disease (13,14,15). The safety and efficacy of cinacalcet in children have been shown in a few reports and in cases of secondary hPTH with disorders such as x-linked hypophosphatemia, HVDRR, and renal failure (15,16). We can only reach the suppressed PTH levels with low-dose cinacalcet combined with high-dose oral calcium and calcitriol therapy. After 4 months of this combination therapy, long-term normocalcemia and PTH suppression with normalization of serum phosphorus level were achieved in addition to radiological healing, but, as expected, there was no recovery in alopecia. Treatment with cinacalcet, as a side effect, may cause hypercalciuria due to activation of calcium-sensing receptors in the thick ascending limb of the loop of Henle, and for this reason, administration of thiazide diuretics may be beneficial (16). Another potential risk of cinacalcet therapy is hypocalcemia due to hypercalciuria. In Case 1, we have observed a temporary hypocalcemia but not hypercalciuria. At the beginning, Case 2 was also treated with high-dose oral calcium and calcitriol, but this proband did not respond to this therapy protocol. We observed only temporary PTH suppression, metabolic and radiological healing with intermittent i.v. calcium infusion (Table 2). Therefore, to achieve long-term metabolic and radiological healing as in Case 1, low-dose cinacalcet was added to the high-dose oral calcium and calcitriol therapy. Both two siblings are at present continuing treatment under the same protocol.

In this report, we described two siblings with HVDDR from parents with consanguinity. Heterozygous mutations in VDR gene were detected in the parents while the children were homozygous for the same mutation. Our patients did not respond to traditional treatment with calcium and calcitriol in high doses, and responded partially to intravenous calcium infusions. In addition to high-dose calcium and calcitriol, by administrating cinacalcet we were able to obtain a positive effect in maintaining the biochemical and radiological healing. Thus, we have shown that cinacalcet improves the biochemical and radiological findings in HVDDR and we therefore recommend its use in the management of secondary hPTH.

## Figures and Tables

**Table 1:**
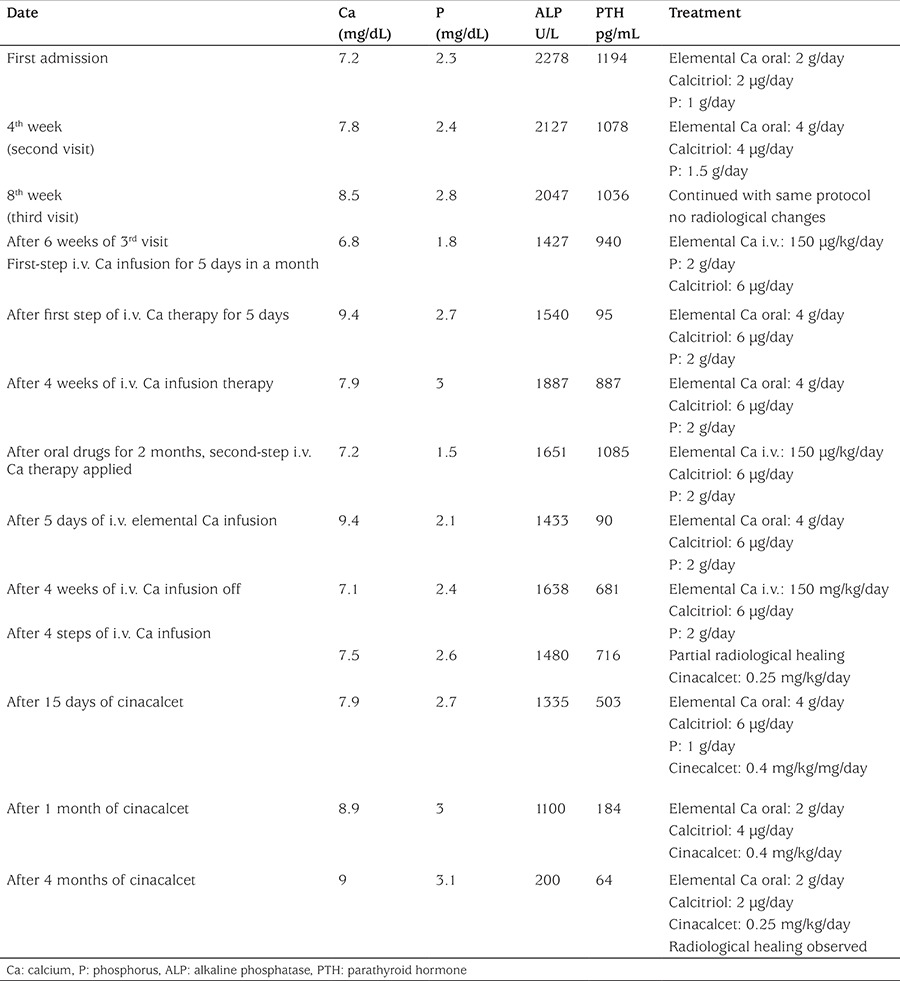
Effects of treatment with elemental calcium (oral/i.v.), calcitriol, and cinacalcet on serum calcium, phosphorus, alkaline phosphatase, and parathyroid hormone levels in case 1

**Table 2:**
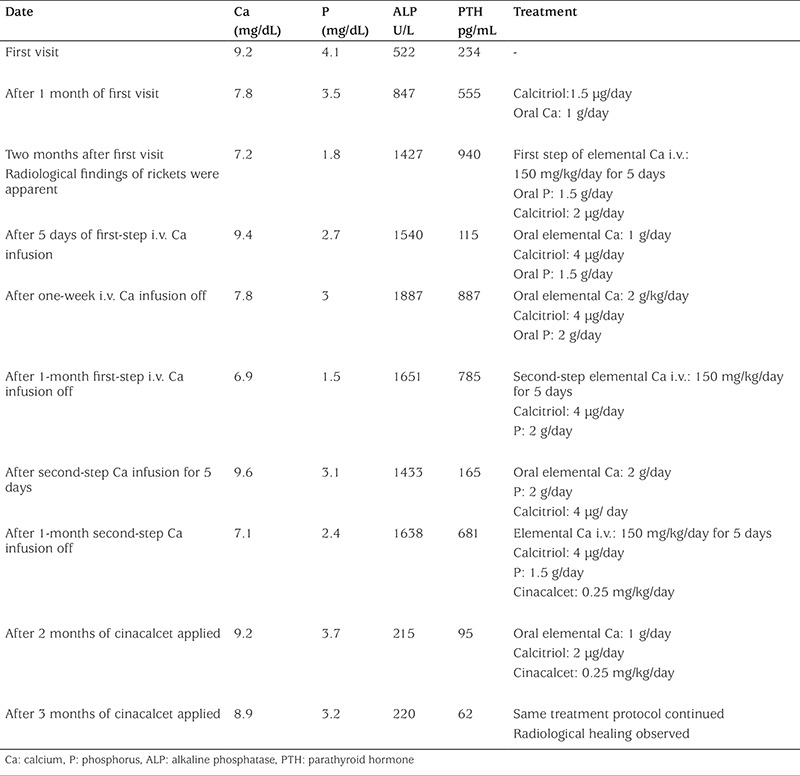
Effects of treatment with elemental calcium (oral/i.v.), calcitriol, and cinacalcet on serum calcium, phosphorus, alkaline phosphatase, and parathyroid hormone levels in case 2
